# A FITM1-Related Methylation Signature Predicts the Prognosis of Patients With Non-Viral Hepatocellular Carcinoma

**DOI:** 10.3389/fgene.2020.00099

**Published:** 2020-02-27

**Authors:** Jie Chen, Xicheng Wang, Xining Wang, Wenxin Li, Changzhen Shang, Tao Chen, Yajin Chen

**Affiliations:** ^1^Department of Hepatobiliary Surgery, Sun Yat-sen Memorial Hospital, Sun Yat-sen University, Guangzhou, China; ^2^Guangdong Provincial Key Laboratory of Malignant Tumor Epigenetics and Gene Regulation, Medical Research Center, Sun Yat-sen Memorial Hospital, Sun Yat-sen University, Guangzhou, China; ^3^Department of Cardiology, The Eight Affiliated Hospital, Sun Yat-sen University, Shenzhen, China

**Keywords:** methylation-driven genes, non-viral hepatocellular carcinoma, FITM1, signature, nomogram

## Abstract

Although great progress has been made in treatment against hepatitis virus infection, the prognosis of hepatocellular carcinoma (HCC) remains unsatisfied. Therefore, there is an unmet need to explore biomarkers or prognostic models for monitoring non-viral hepatocellular carcinoma. Accumulating evidence indicates that DNA methylation participates in carcinogenesis of malignancies. In the present study, we analyzed 101 non-viral HCC patients from TCGA database to figure out methylation-driven genes (MDGs) that might get involved in non-viral HCC pathogenesis using MethyMix algorithm. Then we picked out 8 key genes out of 137 MDGs that could affect the overall survival (OS) of both methylation and expression level. Using PCA, Uni-variate, Multi-variate, and LASSO cox regression analyses, we confirmed the potential prognostic value of these eight epigenetic genes. Ultimately, combined with immunohistochemistry (IHC), ROC, OS, and GSEA analyses, fat storage-inducing transmembrane protein1 (FITM1) was identified as a novel tumor suppressor gene in non-viral HCC and an applicable FITM1-methylation-based signature was built in a training set and validated in a testing set. Briefly, our work provides several potential biomarkers, especially FITM1, as well as a new method for disease surveillance and treatment strategy development.

## Introduction

Hepatocellular carcinoma (HCC) is a highly malignant tumor with high mortality and brings a great burden to social economy (Siegel et al., 2017). Chronic virus infection, commonly hepatitis B virus (HBV) and hepatitis C virus (HCV), and long-term alcohol consumption are the major etiology of HCC development (Braillon, 2012). Thanks for the development of vaccine and anti-virus medication treatment, the morbidity of virus-related HCC shows a decreasing tendency. Although viral hepatitis infection is strongly responsible for liver cancer progression, various non-viral risk factors play important roles in promoting HCC development (Alzahrani et al., 2014). The epidemiological studies show that the incidence of HCC has failed to decline over the past decades partly owing to the increasing of HCC without virus infection. Thus, there is an unmet need to understand the underlying molecular mechanism of non-viral HCC. Due to the high heterogeneity and molecular diversities (Bruix et al., 2014), the prognosis of non-viral HCC patients is widely divergent. Therefore, an effective and accurate model to predict the prognosis of non-viral HCC individually is important and helpful to inform future clinical-decision making.

DNA methylation, one of the predominant forms of pre-transcriptional modification, has been widely studied in carcinogenesis (Noguchi et al., 1994; Chuang and Chiang, 2014). To date, great attention has been paid to investigate the relationship between methylation-driven genes (MDGs) and tumorigenesis (Pu et al., 2017). Recent studies have proved that MDGs participates in various lethal diseases like lung adenocarcinoma (Gao et al., 2018), pancreatic cancer (Gevaert et al., 2015), renal carcinoma (Zhang et al. 2019; Wang et al., 2020), and colon cancer (Chen et al., 2016). Meanwhile, many studies revealed that numerous genes are abnormally hypermethylated or hypomethylated in HCC (Hlady et al., 2019). Therefore, whether MDGs take part in the initialization and progression of non-viral HCC or not remains to be verified and a comprehensive understanding of several potential targets or biomarkers urges to be made.

Since long, methylation has been proved to negatively regulate gene expression, and DNA methylation is accountable for multiple cancers, including HCC (Revill et al., 2013; Hlady et al., 2019). Recently, Sun et al. has revealed that a novel gene signature (CTHRC1 expression, ZIC4 expression, and OTX1expression) may be regulated by DNA hypermethylation and closely associated with HCC through weighted correlation network analysis (WGCNA) (Sun et al., 2018). A 21-gene pairs signature was established to predict HCC patients at their early stages through the C-index forward search method (Liu et al., 2018). In addition, six MDGs, including SNHG6, S100P, DCDC2, LIME1, FMO3, and GPR171, have been selected to construct a predictive signature for HCC patients and the contribution of virus infection has been highly emphasized in their work (Li et al., 2019). Wang et al. also constructed a risk score system consisting of BRCA1 expression, CAD expression, RBM8A expression and CDC20 expression by using four GSE data (Wang et al., 2019). However, a novel methylation prognostic signature for non-viral HCC still remains undeveloped and a systematic exploration of non-viral HCC signature is needed. To our knowledge, the eight MDGs studied in present work has never been recruited to be part of a score signature in HCC, indicating that they might exert important effect on the tumorigenesis and development of non-viral HCC specifically.

In current study, we utilized an integrative method, including MethyMix tool, principal component analysis (PCA), nomogram algorithm, and least absolute shrinkage and selection operator (LASSO) regression analysis, to explore prognosis related to MDGs in non-viral HCC and validate the efficacy of the built methylation-related risk signature, providing a novel direction for treatment and surveillance strategy and personalized follow-up for non-viral HCC patients.

## Methods and Materials

### Data Processing and Analysis

The RNA-seq data, methylation data, and corresponding clinicopathological information were retrieved for 101 non-viral HCC patients from TCGA database. Clinicopathological features for the TCGA datasets were described in **Supplementary Table S1**. On the basis of the MethylMix algorithm (Gevaert, 2015; Cedoz et al., 2018), we analyzed the correlation between gene methylation and expression level in 121 non-viral HCC samples. Due to the strict constraints of MethylMix algorithm, we set the parameters as followed: Adjust *P*-value< 0.05; Log FC (Fold Change) > 0 or Log FC< 0; Pearson correlation threshold< -0.3. Then, we identified aberrantly hypomethylated or hypermethylated genes by constructing the β-mixed model. Finally, according to the overall survival analysis results, we filtered most MDGs and obtained several key genes for further study. The mRNA expression and methylation data of non-viral HCC provided by TCGA is open-access and the approval of a local ethics committee is unneeded.

### Gene Ontology (GO), Disease Ontology (DO), and KEGG Pathway Enrichment Analyses

In the present study, the clusterProfiler package (version 3.12.0) was applied to conduct GO and Kyoto Encyclopedia of Genes and Genomes (KEGG) pathway enrichment analyses (Yu et al., 2012). The GO analysis includes cellular composition (CC), molecular function (MF), and biological process (BP). Disease ontology (DO) annotates genes based on human disease. DO is vital annotation, translating obtained key genes to clinical relevance. And DOSE, an R package, is capable of analyzing semantic similarity computations of the DO terms and genes. Therefore, DOSE enables us to figure out the closeness between diseases and gene functions (Yu et al., 2015). To investigate the underlying mechanism of these MDGs, 137 MDGs were subjected to clusterProfiler and DOSE packages for GO, KEGG, and DO analyses; and *P*-value < 0.05 was set as the cutoff.

### Kaplan-Meier Curves of MDGs and Methylated Sites

For the sake of studying the prognostic evaluation of MDGs, the survival R package (version3.5.1) was used to calculate the prognostic survival analysis of the gene expression, gene mean methylation level and the methylated sites, performed by integrating the clinical data and prognostic information of non-viral HCC in TCGA. Meantime, we conducted a joint survival analysis of gene methylation and expression levels to further determine key genes associated with prognosis in non-viral HCC patients.

### PCA Analysis and Subgroup Analysis

To study the function of eight methylation-driven key genes in non-viral hepatocellular carcinoma, we separated 101 non-viral HCC patients into different subgroups by the approach of “ConsensusClusterPlus”, an algorithm for determining clusters by the unsupervised analysis based on gene expression (Wilkerson and Hayes, 2010). The consensus clustering tool presents measurable and visible evidence to estimate the counts of unsupervised classes in a dataset. The maximum evaluated k (max K) is 9 and other parameters of ConsensusClusterPlus are default. As a result, Subgroup 1 had 59 non-viral HCC patients while Subgroup 2 had 41 patients. And when the data were classified into three subgroups, there were 33, 36, and 31 patients in Subgroup 1, 2, and 3, respectively. The R package (R v3.5.1) of PCA analysis was adopted to explore the gene methylation patterns in subgroups of non-viral HCC. Gene Sets Enrichment Analysis (GSEA) was performed by GSEA 4.0.0 software to explore the specific KEGG pathways related to distinct subgroups of non-viral HCC and the underlying function of FITM1 (Subramanian et al., 2005). Regarding the GSEA results, |NES|> 1 and *P*-value < 0.05 were considered significant in our study.

### Construction of MDGs Signature

Caret R package (Classification and Regression Training; Version:6.0-84) can provide a wide variety of predictive models by integrating more than 25 other relative packages and has various unique features such as data splitting, characterizing performance, pre-processing, parallel processing, and variable importance (Kuhn, 2008). Due to the lack of other datasets with integrative data of non-viral HCC patients (epigenomics, transcriptomics, and clinical pathologic data), we stochastically divided the 101 non-viral HCC patients into 2 sets, training set (52 patients), and testing set (49 patients). The classification was based on the caret R package. To confirm the prognostic value of 8 MDGs, the Uni-variate cox regression, LASSO cox regression, and Multi-variate cox regression algorithms were performed in the training set and a potential risk signature of non-viral HCC was developed (Bøvelstad et al., 2007; Qiu et al., 2017; Wang et al., 2019). The risk score for the signature (Lossos et al., 2004) was computed using the formula:

$$risk\;score=\;\sum^{}\begin{array}{c} n\\ i=1 \end{array}Coef_{i}*X_{i}$$

As the formula shown above, “X” represents the methylation level of each methylation-driven gene in the samples; The “Coef” means corresponding Multi-variate cox regression coefficient of each factor in the prognostic model. The value of “n” in our study is smaller than 8. On the other hand, a nomogram of non-viral HCC patients was constructed based upon the results of the LASSO cox regression analysis using rms package (version 3.5.1). The prognostic risk value of each patient was calculated using the formula, and the median of the score value was cut off. The non-viral HCC patients were classified into high and low risk groups. Then, we conducted ROC curve and Kaplan-Meier survival curve analyses to validate the signature in both the training set and testing set. Log-rank test was applied to figure out the difference of overall survival rate between the high-risk and low-risk groups. “*P* < 0.05” was considered statistically significant.

## Results

### Identification and Functional Analyses of MDGs in Non-Viral Hepatocellular Carcinoma

**Figure 1:**
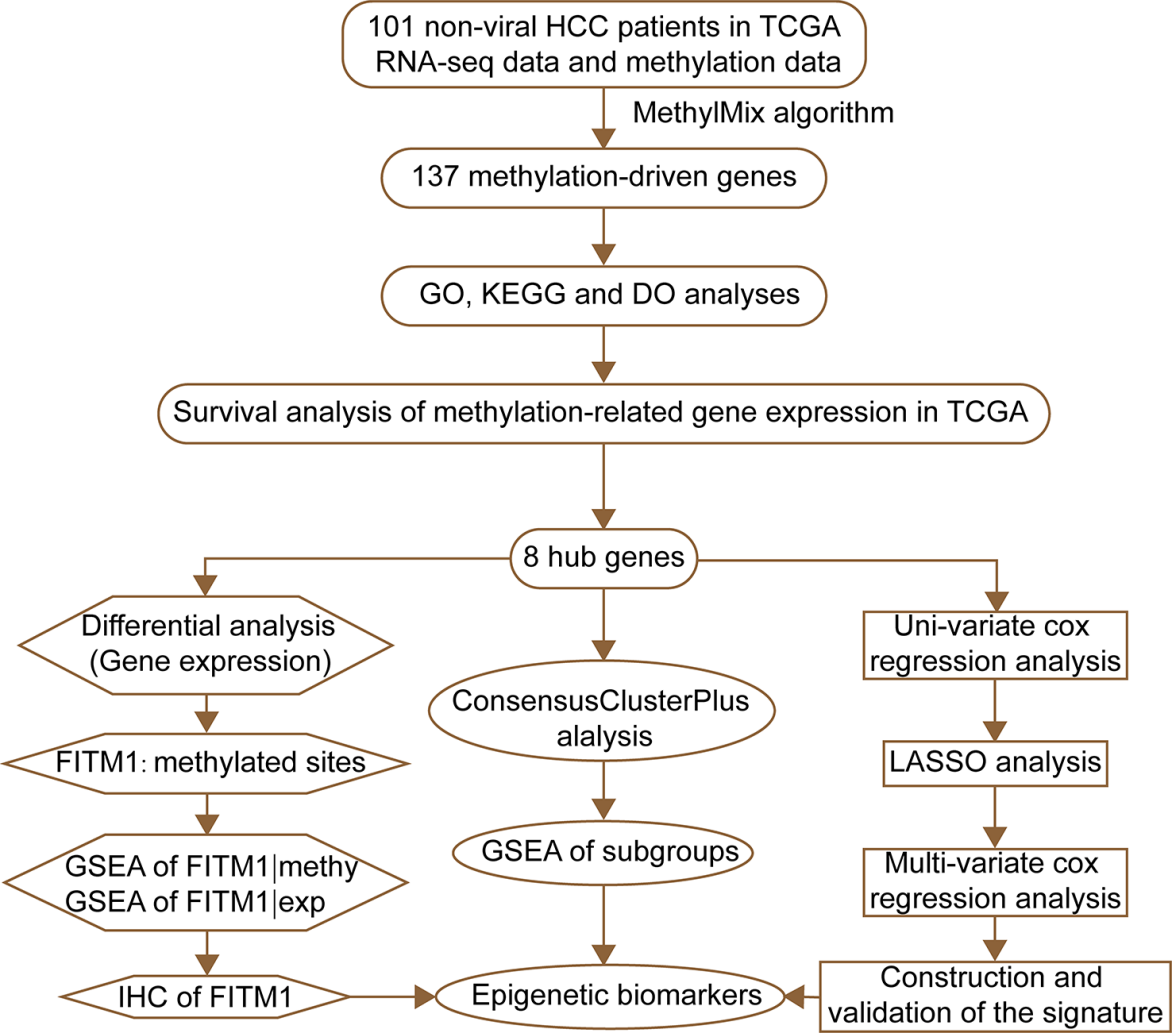
The flowchart of this study.

**Figure 2:**
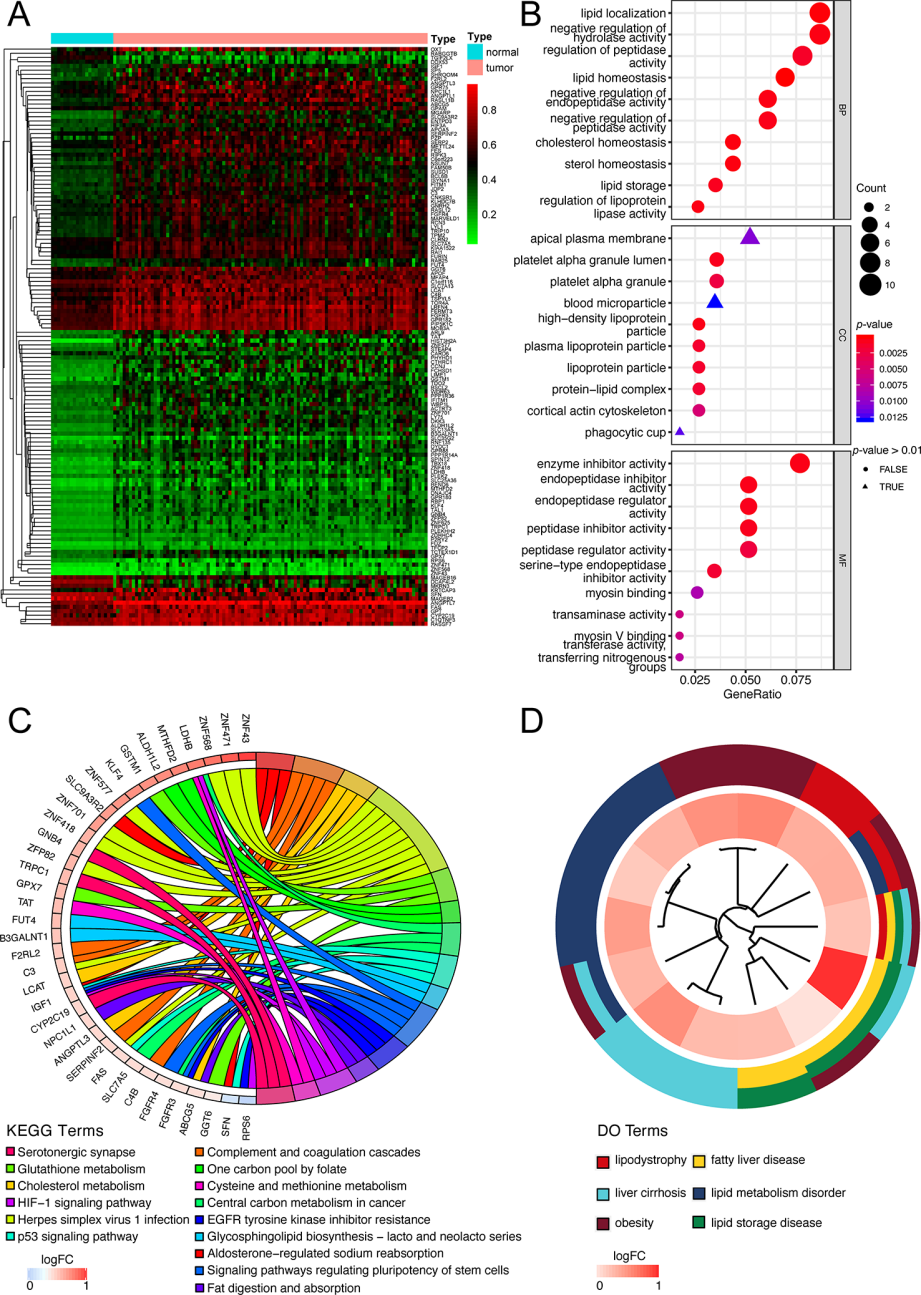
Functional exploration of MDGs. **(A)** Heatmap of 137 aberrant MDGs in non-viral hepatocellular carcinoma. The green color stands for hypomethylation while the red shows hypermethylation. **(B)** Gene Ontology (GO) analysis of 137 MDGs. Only top 10 terms of BP, CC, and MF were shown and the complete data were in **Table S3**. **(C)** KEGG pathway analysis of 137 MDGs. The color of curves represents different KEGG terms; The left semi-circle color means different gene expression and the corresponding genes are labeled. The *P*-value of all terms is < 0.05. **(D)** Disease Ontology (DO) analysis of 137 MDGs. The inner circle is composed of different genes and their expression (LogFC) while the outer circle consists of different DO terms. The *P*-value of shown DO terms is < 0.05. Only liver-related terms were exhibited and the whole results were in **Table S5**.

The flow diagram for present study was exhibited in **Figure 1**. After downloading the comprehensive data of 101 non-viral hepatocellular carcinoma patients, the MethylMix algorithm mentioned above was adopted to figure out 137 MDGs in non-viral HCC (**Figure 2A** and **Table S2**). To elucidate the potential function of these genes, GO, KEGG, and DO analyses were carried out. As shown in **Figure 2B**, the GO top significant terms were various, and some of them were as followed: “lipid localization”, “cholesterol homeostasis”, “lipid homeostasis”, “sterol homeostasis”, “lipid storage regulation of lipoprotein”, “lipoprotein particle”, and “protein-lipid complex”, which indicated that the aberrant methylation level of 137 MDGs may cause abnormal lipid metabolism, one of the most pivotal function of normal liver. In addition, KEGG analysis revealed that these 137 MDGs were significantly enriched in pathways in “Glutathione metabolism”, “Aldosterone-regulated sodium reabsorption”, “Fat digestion and absorption”, and “Cholesterol metabolism”, consistent with the result of GO analysis. “p53 signaling pathway”, “HIF-1 signaling pathway”, and “EGFR tyrosine kinase inhibitor resistance” were also enriched, suggesting the potential regulating signaling pathway of non-viral HCC by MDGs (**Figure 2C**). In addition, for the sake of investigating the relationship between137 MDGs and human diseases. DO analysis was applied. As shown in **Figure 2D**, these genes might be involved in the following DO terms: “lipodystrophy”, “fatty liver disease”, “liver cirrhosis”, “obesity”, and so on. Complete data of the enrichment analyses above were displayed in **Tables S3**, **S4**, **S5**. Taken together, these results indicate that 137 MDGs might participate in the carcinogenesis of non-viral HCC through the regulation of liver lipid metabolism and chronic liver injury.

### Screening and Verifying Survival-Related Key Genes Among 137 MDGs

**Figure 3:**
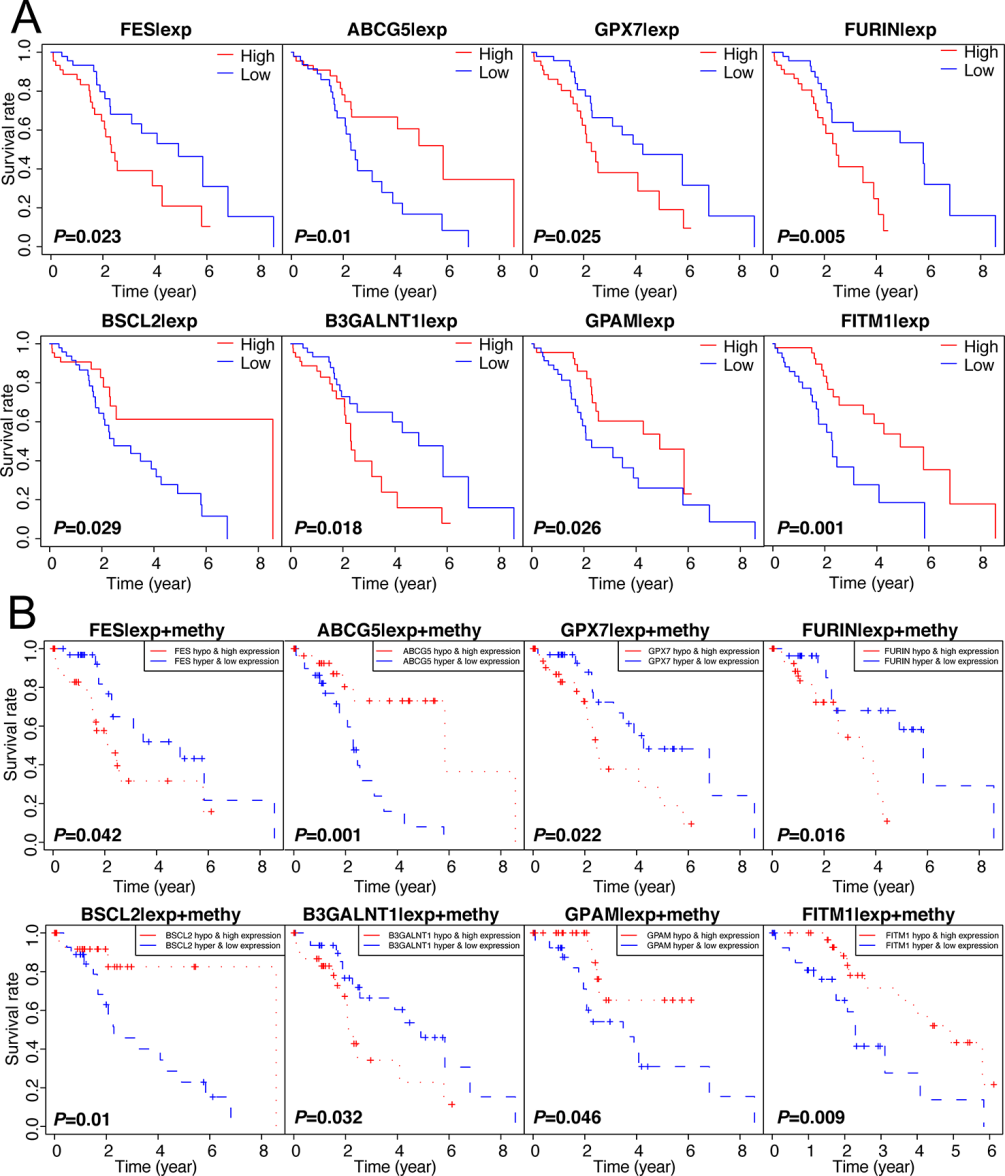
Survival analysis of 137 genes and selection of 8 methylation-driven key genes. **(A)** 137 genes were analyzed by survival analysis in 101 non-viral HCC specimens and only eight key genes were obtained according to the specific cut-off (*P*-value < 0.05). **(B)** Joint survival analysis of 137 oncogenes. we performed survival curve analysis based on the combination of methylation level and expression level and the prognosis-related gene were selected (*P*-value < 0.05).

Figuring out some oncogenes, which act importantly on hepatocarcinogenesis as well as the progression of non-viral HCC, is of great significance. Therefore, we performed not only the overall survival analysis of gene expression but also the joint survival analysis, which analyzes the OS combining the level of expression and corresponding methylation together, across 137 genes in 101 non-viral HCC patients. Thereafter, discarding those results without significant difference (*P*-value > 0.05) from both expression OS and joint OS, eight key genes were selected for further study: FITM1, FES, ABCG5, GPX7, FURIN, BSCL2, B3GALNT1, and GPAM. As shown in **Figure 3A**, low expression of FITM1, ABCG5, BSCL2, and GPAM in non-viral HCC tumor specimens generally predicted worse survival status. However, the high expression of FES, GPX7, FURIN, and B3GALNT1 leaded to shorter survival time. On the other hand, as presented in **Figure 3B**, the hypomethylation and high expression of FES, GPX7, FURIN, and B3GALNT1 were related to poorer overall survival while the adverse results could be obtained from the survival curves of FITM1, ABCG5, BSCL2, and GPAM. In all, these results reveal that the methylation and expression level of these epigenetic genes could affect the prognosis of non-viral HCC patients.

### Abnormal DNA Methylation of Eight Key Genes in Tumor Tissues Negatively Regulated Gene Expression

**Figure 4:**
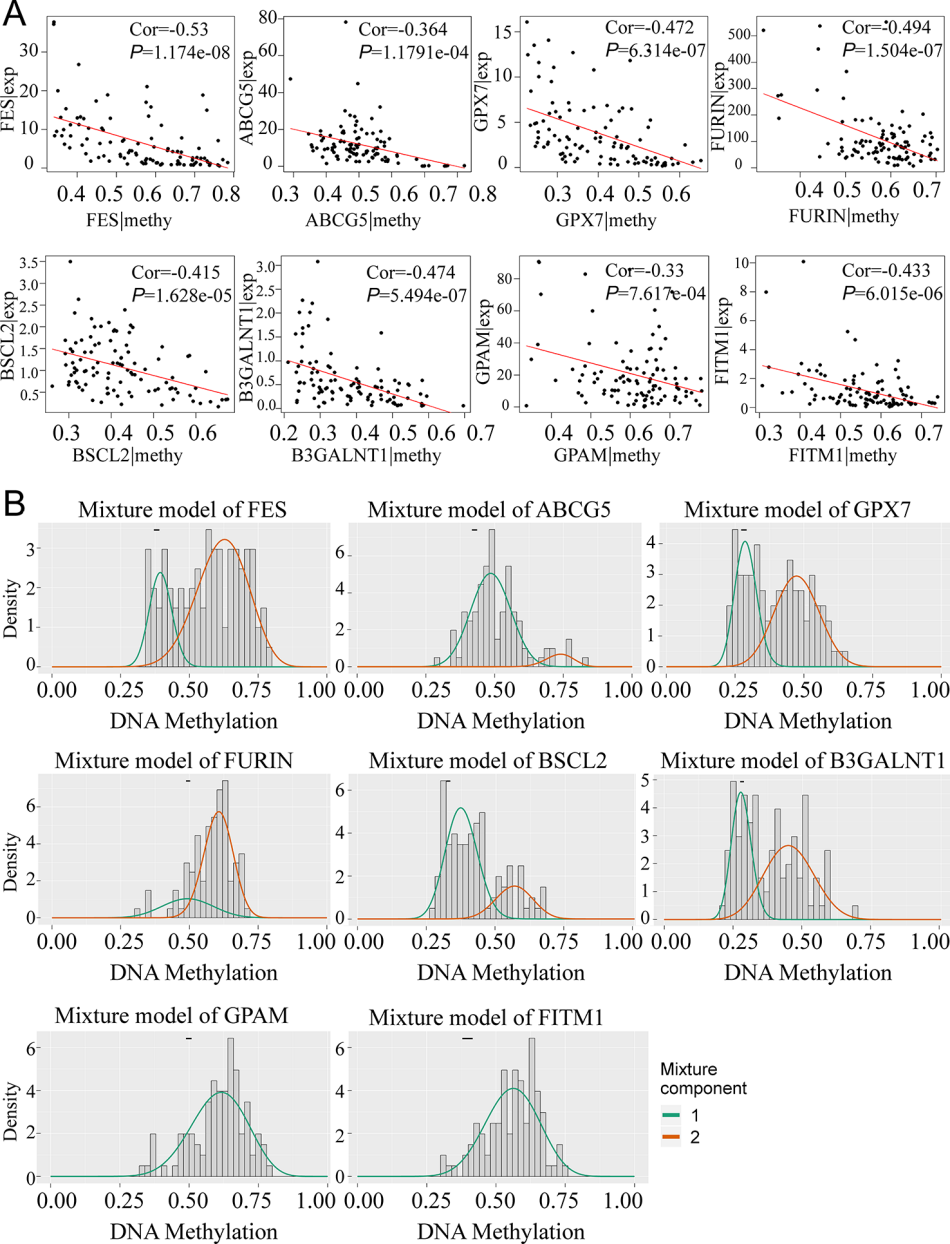
The relationship between eight genes expression and methylation level in non-viral HCC patients. **(A)** The correlation between gene expression and methylation. **(B)** MethylMix model of eight DNA methylation-driven key genes. The abscissa is the methylation degree and the ordinate is the number of sample. The histogram exhibits the distribution of methylation in 101 non-viral HCC samples and the short black bar above the histogram is the methylation distribution of 20 normal tissues. The classification of different methylation degree of the malignancies relative to the normal tissues can be distinctly observed from the figures.

To obtain a deeper understanding of DNA methylation and mRNA expression of eight key driver genes, correlation analyses between methylation level and gene expression were employed. According to **Figure 4A**, along with the increase of the methylation degree, the key gene expression showed a downward trend, suggesting the negative correlation between DNA methylation and gene expression. Notably, we found that the aberrant methylation degree was much higher in tumor rather than normal tissues. Significantly, only 1 methylation curve of FITM1 or GPAM was gathered, indicating that the hypermethylated status of FITM1 and GPAM were centralized and common in tumor samples (**Figure 4B**). And FES, ABCG5, GPX7, FURIN, BSCL2, and B3GALNT1 had 2 methylation curves and the comparison of methylation level in tumors and normal tissues was ambiguous, driving us to elucidate the gene methylation and expression level between malignant samples and normal samples in non-viral HCC patients.

### FITM1 Was Significantly Down-Regulated in Non-Viral HCC and Was a Potential Tumor Suppressor Gene

**Figure 5:**
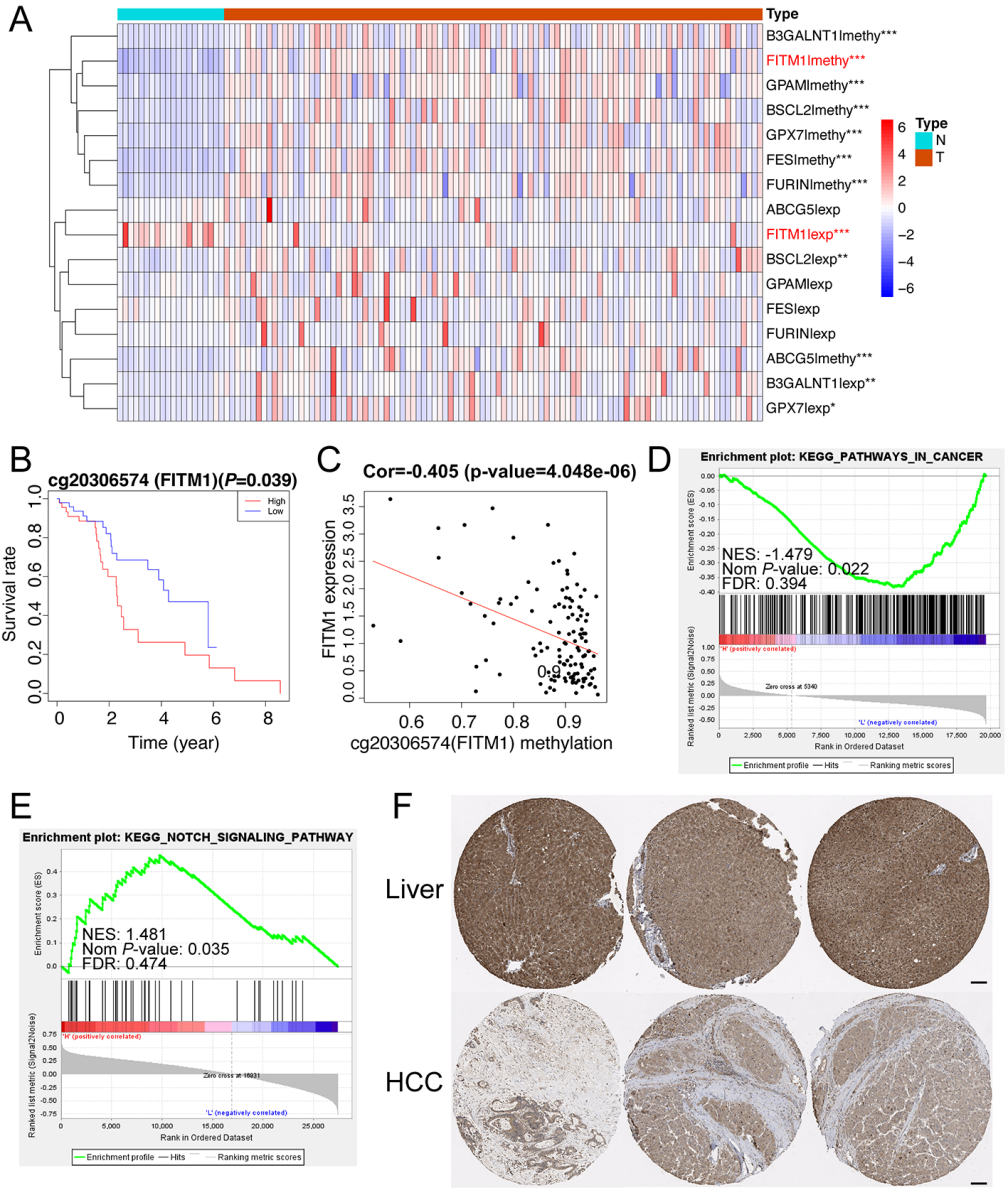
Function and mechanism of FITM1 in non-viral HCC. **(A)** The thermal map of eight gene expression levels and eight gene methylation levels. The differential analysis between 20 normal samples and 101 tumor samples were conducted by Limma R package (version: 3.42.0). “*”, “**”, and “***” stands for “*P*-value < 0.05”, “*P*-value < 0.01”, and “*P*-value < 0.001”, respectively. The thermal map was drawn with the pheatmap R package (version: 1.0.12) and the row scale (Z-score) was chosen to better visualize the related data. **(B)** The correlation of FITM1-related CpG methylated site and its gene expression. **(C)** The survival analysis of FITM1-related CpG methylation. **(D)** The GSEA result based on FITM1 expression level in 101 non-viral HCC patients. **(E)** The GSEA result based on FITM1 methylation level in 101 non-viral HCC patients. **(F)** The IHC of FITM1 retrieved from the Human Protein Atlas database (https://www.proteinatlas.org). Scale bars:100um.

To illustrate the clear distribution and different expression of eight key genes in the normal and tumor samples of non-viral HCC, a thermal map was performed based on the gene methylation and corresponding expression. As vividly shown in **Figure 5A**, we found that FITM1, BSCL2, B3GALNT1, and GPX7 also had significantly different expression in comparison tumors with normal specimens. However, only FITM1 expression was significantly down-regulated in tumor specimens (Log FC = -1.74, *P*-value = 8.75E-09) while other three were over-expressed. Moreover, the FITM1 methylation was highly up-regulated in tumor specimens (Log FC = 0.49, *P*-value = 2.00E-09). This significantly negative correlation between methylation and expression in tumor as well as normal tissues triggered us to further explore the specific function of FITM1. For the sake of identifying the most worthwhile MDGs related to prognosis of non-viral HCC, we explored the characteristics of CpG methylated sites of FITM1. The methylation degree of cg20306574 methylated sites was negatively correlative with FITM1 expression level (**Figure 5B**). Besides, high cg20306574 methylation predicted poorer prognosis in 101 non-viral HCC patients (**Figure 5C**). To investigate the anti-tumor mechanism of FITM1, the GSEA analysis was applied to analyze the methylation data and the mRNA expression data of 101 non-viral HCC patients in TCGA. As displayed in **Figure 5D**, we figured out that low FITM1 expression could activate cancer-related pathway. In addition, several metabolism-related signaling pathways might be able to account for the anti-tumors effect of FITM1, indicating the underlying function of FITM1 in non-viral HCC (**Table S6**). Regarding the gene methylation level of FITM1, one of the enriched results pointed toward the NOTCH signaling pathway. Moreover, several lipid-related metabolism pathways were also enclosed (**Table S7**), partly consistent with the GO results of 137 MDGs in **Figure 2B**. As for the immunohistochemistry results (IHC) of FITM1 obtained from the Human Protein Atlas database (https://www.proteinatlas.org) (Uhlen et al., 2015), **Figure 5F** vividly shows that FITM1 expression was much higher in normal liver tissues rather than HCC tissues. Briefly, these aforementioned results indicate that FITM1 is closely related to non-viral HCC.

### ConcensusCluster Analysis Revealed That Cluster 1 Might Regulate the NOTCH and TGF-Beta Signaling Pathways

**Figure 6:**
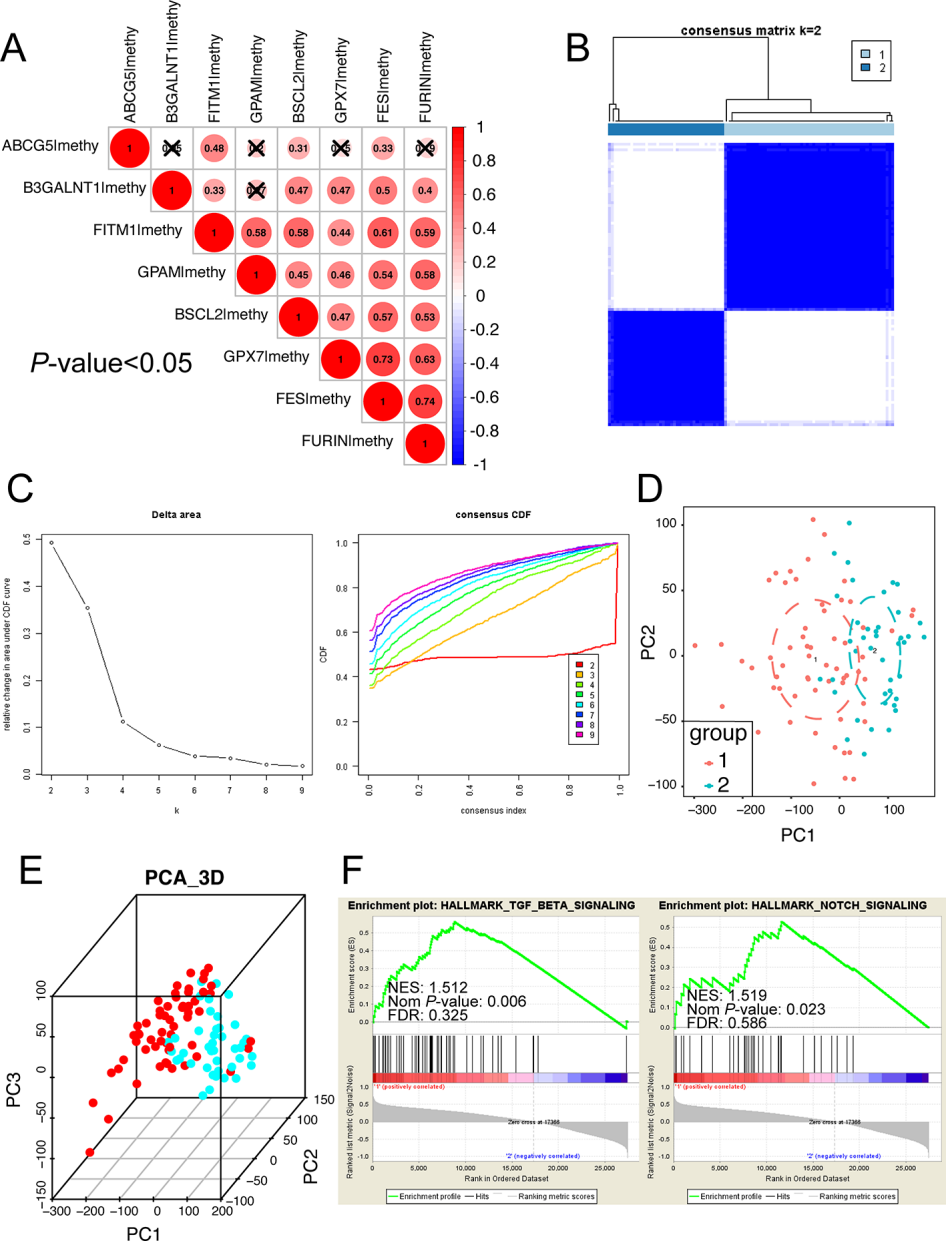
Subgroup analysis developed on the methylation level of eight key MDGs. **(A)** The methylation relationship among eight key genes. The bigger the circle size, the more correlative two genes are. **(B)** Consensus matrix of two subgroups (k = 2). The k = 3 to 10 of the consensus matrix were shown in **Figure S1**. This study distinctly separated the whole methylation data into two subgroups: cluster 1 and cluster 2. **(C)** Classification of consensus clusters by 8 key MDGs. Consensus clustering cumulative distribution function (CDF) was set from k = 2 to 10. **(D)** Principal component analysis (PCA) of the total methylation level in 101 non-viral HCC patients based upon the consensus clustering. Non-viral HCC patients in different clusters are noted with different colors. **(E)** The 3D PCA of two subgroups according to 101 non-viral HCC patients. The 3D PCA of three subgroups were displayed in **Figure S1**. **(F)** The significant GSEA analysis results of cluster 1. The full GSEA data of clusters were included in **Table S8**.

Considering that the eight survival-related MDGs might exert its regulatory effect on non-viral HCC synergistically, we performed the correlation and subgroup analysis among the methylation level of eight key genes. As shown in **Figure 6A**, the methylation degrees of eight key genes were positively relative to each other, especially FITM1, BSCL2, and FES, which were significantly correlated with other seven key genes respectively. Furthermore, ConcensusCluster analysis was utilized to classify the tumor samples based upon the methylation level similarity of the eight MDGs. As revealed in **Figure 6B** and **Figure S1A**, k = 2 was selected as a reasonable choice with cluster stability rising from k = 2 to 10 in the non-viral HCC dataset. However, the CDF curve revealed that k = 3 was also preferable (**Figure 6C**). Therefore, we used PCA analysis to investigate the characteristic of methylation profile based on the classification of both k = 2 and k = 3 in 101 non-viral HCC patients. The results exhibited an evident difference between 101 non-viral HCC patients in both the 2D and 3D plotting of PCA results according to the two-subgroup classification (**Figures 6D, E**). And the two-subgroup classification could also well distinct tumor samples from normal samples (**Figures S1D, E**). Meantime, as illustrated in **Figures S1B, C**, 3-subgroup classification of 101 non-viral HCC patients was also capable of classifying non-viral HCC patients while the overlaid part was more than two-subgroup classification (**Figures S1B, C**). In all, the classification built on the methylation level of eight key genes might be more distinguishable when the non-viral HCC patients were divided into two subgroups. In addition, the GSEA analysis was further conducted to explore the hallmark of two-subgroup classification. As presented in **Figure 6F**, we found that Cluster 1 was closely related to NOTCH and TGF-beta signaling pathways, both playing vital roles in malignancies. In all, we demonstrate that the carcinogenesis of patients in Cluster 1, rather than Cluster 2, might be involved in NOTCH and TGF-beta signaling pathways.

### A Risk Signature Was Established in Training Set Using Three Selected Key Genes Related to DNA Methylation and Prognosis

**Figure 7:**
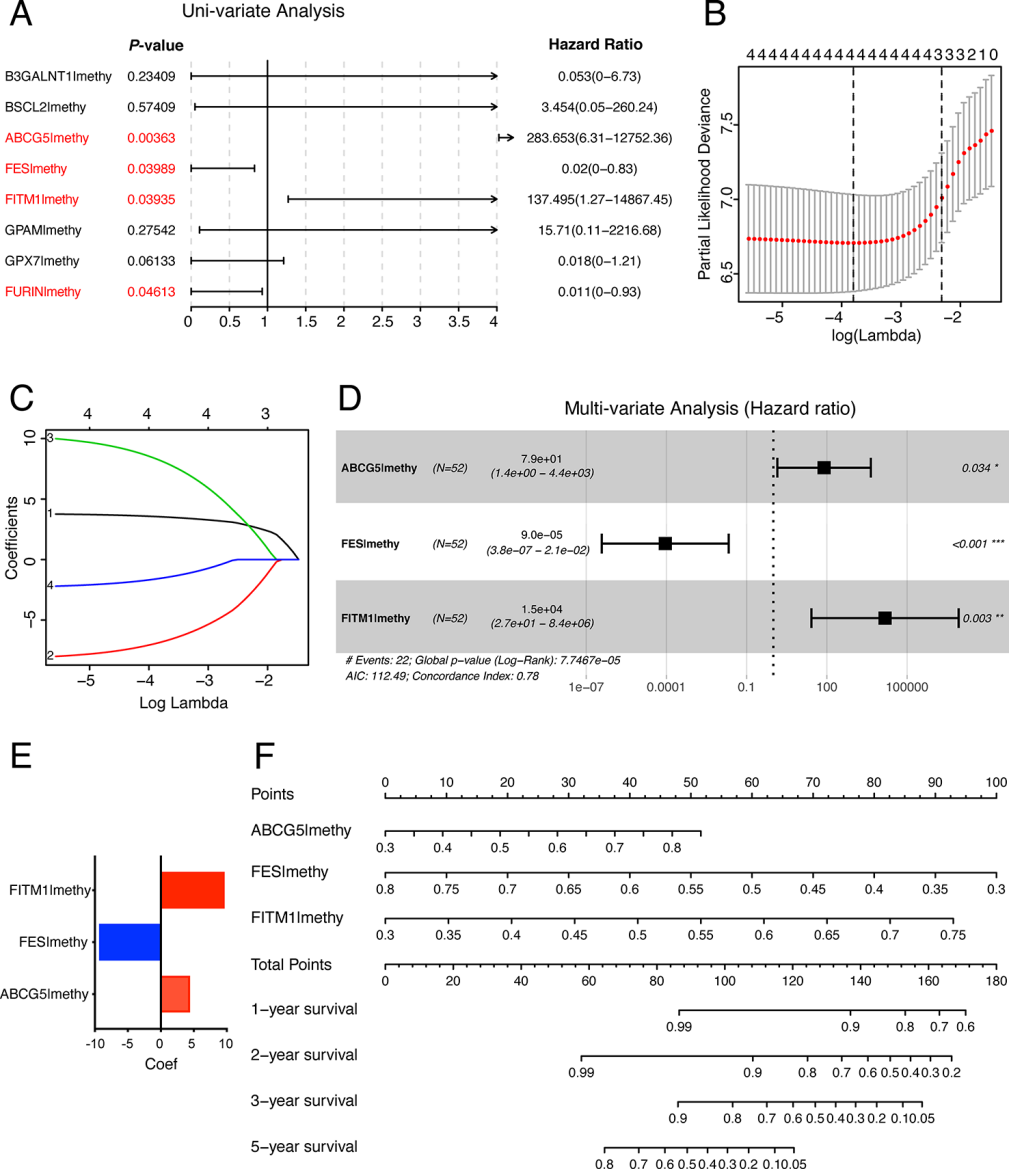
The construction of the score model in training set**. (A)** Uni-variate analysis of eight key MDGs in the training set of non-viral HCC patients. The genes in red (*P*-value < 0.05) were selected and imported into the LASSO algorithm to conduct risk score model for non-viral HCC patients. **(B**, **C)** LASSO analysis of four selected genes in training set. **(D)** Multi-variate analysis of four selected genes in training set and only 3 out of 4 were significantly imported into the score model. **(E)** Our risk score formula obtained from discovery cohort was as followed: risk score = 4.37 * methylation of ABCG5- 9.31 * methylation of FES + 9.61 * methylation of FITM1. **(F)** Prediction of OS in non-viral HCC based upon nomogram. Three factors were included in this nomogram. The methylation level of these four genes could be used to create points according to the scale plotted upward. And the total points could point to the corresponding probability of 1-year, 2-year, 3-year, and 5-year OS rate drawn on the three lines below.

In order to establish and validate a risk signature for non-viral HCC patients, Caret R package (Version:6.0-84) were conducted to randomly allocate 101 non-viral HCC patients into two sets: training set and testing set. As a result, 52 non-viral HCC patients were included in the training set while 49 patients in the testing set. To better predict the clinical outcomes of non-viral HCC with the eight MDGs, we used the Uni-variate, LASSO, and Multi-variate cox regression algorithm to build the risk signature according to the minimum criteria. As shown in **Figure 7A**, four out of eight key genes were chosen as risk factors using Uni-variate analysis (*P*-value< 0.05). In order to further confirm the result of Uni-variate analysis, these 4 genes were imported into the LASSO algorithm. **Figures 7B, C** show that both 4 or 3 key genes were reliable to construct a risk signature. Finally, Multi-variate analysis was used to construct the risk signature and only three risk factors were significantly chosen (**Figure 7D**). The coefficients of risk factors were retrieved from the Multi-variate analysis and the formula was as followed: risk score = 4.37 * methylation of ABCG5- 9.31 * methylation of FES + 9.61 * methylation of FITM1 (**Figure 7E**). Notably, we found that FITM1 was also recruited in the risk signature (**Figure 7E**), indicating the pivotal role of FITM1 in non-viral HCC. To further explore a driver genes model that could serve as an independent prognosticator for non-viral HCC patients, a visualized and applicable nomogram was built based on three key genes selected by cox regression analyses applied above (**Figure 7F**).

### Prognostic Risk Scores Exhibited Strong Predictive Power in the Prognosis of Non-Viral HCC Patients in Both Training Set and Testing Set

**Figure 8:**
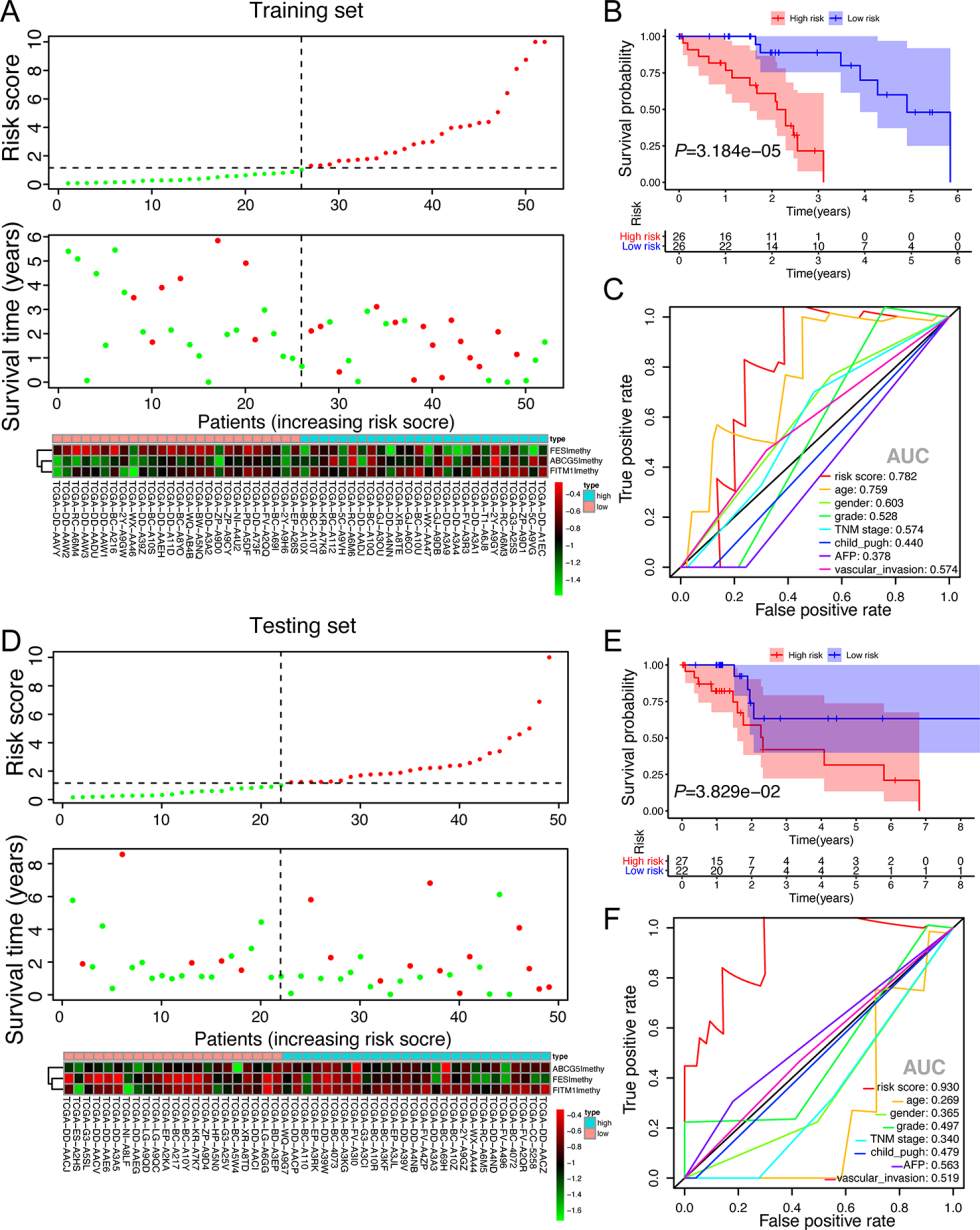
The validation of the score model in training set and testing set. **(A**, **D)** Risk score distribution of non-viral HCC patients, survival curve and methylation heatmap of the three factors of the score model corresponding to each patient in training set (52 patients) and testing set (49 patients). The risk score was all calculated by the score model built on training set. Red color stands for high risk and hypermethylation; blue color means low risk and hypomethylation. **(B**, **E)** Kaplan-Meier survival analysis of the low and high-risk group. **(C**, **F)** ROC curves for a risk score model and several complete clinicopathological information of non-viral HCC patients retrieved from TCGA.

To better understand the function of this risk signature, **Figures 8A, D** were plotted and it could show the explicit relationship between the risk score, survival status, and methylation level in training set and testing set. Not only did the high-risk group in training set have significantly worse OS than the low risk group, the high-risk group in testing set presented the similar phenomenon according to the survival curve in **Figures 8B, E**. In order to find out whether the risk signature was an effective prognostic indicator, receiver operating characteristic curve (ROC) was plotted. The ROC curves showed that the risk score model was able to predict 5-year survival rates for non-viral HCC patients both in training set (AUC = 78.2%) and in testing set (AUC = 93.0%). The predictive power of this model was better than other clinicopathological features included (**Figures 8C, F**). These results suggest that the risk score developed from three key genes could independently predict prognosis in non-viral HCC.

## Discussion

Virus infection is considered as one major cause of hepatocarcinogenesis (Lee et al., 2019). With the rapid development of anti-viral therapies, the virus-related HCC, especially HBV and HCV, is reducing all over the world. However, the incidence of hepatocellular carcinoma remains high and the recurrence rate still render it one of the most lethal malignancies. Other factors like diabetes, non-alcoholic steatohepatitis, non-viral hepatitis, tobacco smoking, obesity, and dietary exposures are accountable for the etiology and progression of non-viral HCC (Dhanasekaran et al., 2016; Ogunwobi et al., 2019). And other potential gene-related causes of non-viral HCC, including TNFα, IL6, mTOR, MAPK, and NF-κB, are garnering close attention (Alzahrani et al., 2014). However, the specific mechanism of non-viral HCC is still unclear. It is of importance to figure out potential biomarkers, score signatures, and even the underlying specific targets to identify, monitor and treat non-viral HCC patients.

In our work, we downloaded methylation and corresponding expression data in TCGA database to explore and retrieve about 137 MDGs by MethyMix analysis. Enrichment analyses of the 137 MDGs indicated they participated in principal biological processes of liver, including lipid metabolism, cholesterol metabolic process, and lipid homeostasis. Given that disorder lipid metabolism is closely associated with non-alcoholic fatty liver disease (NAFLD) and has been growingly considered a hallmark of cancer cells (Cingolani and Czaja, 2016; Tian et al., 2019), It is reasonable that MDGs exerted great impact on non-viral HCC through regulation of lipid metabolism and homeostasis. In addition, KEGG analysis suggested that EGFR and P53 pathways were also significantly enriched, which were involved in the pathogenesis and tumorigenesis of HCC (Xie et al., 2018; Huang et al., 2019).

Based on gene expression overall survival and joint overall survival analysis, we figured out eight key genes. B3GALNT1 is involved in the tumorigenesis of lung adenocarcinoma (Aubry et al., 2015); BSCL2 is over-expressed in the better progression-free and overall survival group of high-grade serous ovarian cancer (Cuello et al., 2017) and may take part in regulating lipid storage in adipocytes and inhibiting ectopic lipid droplet formation in cancer cells (Salo et al., 2016); ABCG5, which could regulate the transport of hydrophobic mixtures, especially lipids, across cellular membranes, is hypermethylated in prostate cancer (Kerr et al., 2011; Devaney et al., 2015); FES hypermethylation and low protein expression were correlated with the PFS (progression-free survival) and OS in HCC (Zhang et al., 2019). As for FITM1, previous study showed that knocking out the FITM1, the lipid droplet accumulation reduces, suggesting that the expression of FITM1 has a connection with lipid droplet, which has a great impact on inflammation, metabolic disorders, and cell injury in liver (Goh and Silver, 2013). Moreover, FITM1 is a member of evolutionarily conserved gene family found in 2008, which plays an important role in fat storage (Kadereit et al., 2008). It closely relates to PPARα in an organ specific way and commonly express at a low level in liver compared with other organs like heart and skeletal muscle in mammals (Rodriguez and Kersten, 2017). Given that FITM1 belongs to a protein family with unique structure and involves in the key progress of lipid metabolism, the aberrant methylation state of FITM1 might result in disorder lipid homeostasis and NAFLD (Gross et al., 2011; Goh and Silver, 2013), triggering the carcinogenesis and progression of the non-viral HCC.

In our study, we revealed that FITM1 expression was much lower in tumor tissues compared with other seven key genes or corresponding normal samples. We also suggested that hypermethylation of FITM1 might account for the downregulation of FITM1 expression partly through modulating NOTCH signaling pathway. More interestingly, as displayed in **Figure S2A**, we found that FITM1 was also down-regulated in the whole HCC patients in TCGA with or without viral infection according to the GEPIA database (http://gepia.cancer-pku.cn) (Tang et al., 2017). And the expression of FITM1 was negatively correlated with the TNM stage (**Figure S2B**). Moreover, low expression of FITM1 predicted worse prognosis in HCC patients (**Figures S2C, D**). However, the specific molecular function of these key genes in HCC, principally FITM1, is still ambiguous. Though the exploration of FITM1 in silico strongly indicated that FITM1 hypermethylation participated in the progression of non-viral HCC by silencing FITM1 expression and it could act as a tumor suppressor gene, related experiments of FITM1 expression, and FITM1 methylation in non-viral HCC still need to be conducted *in vitro* and *in vivo* in the continued study.

While the efficacy of any single biomarker is inadequate, a multiple-risk signature might exert much greater prognostic value for non-viral HCC patients. Therefore, a FITM1-related signature was established in training set through Uni-variate, LASSO, and Multi-variate cox regression analyses and the validation was performed by survival curve and ROC curve analyses in training set and testing set. To make it suitable for the clinical context, we then constructed a nomogram to judge the prognosis of non-viral HCC patients directly and visually. The risk signature and nomogram could enable doctors to identify high and low risk non-viral HCC patients, delivering helpful evidences to make better individualized treatment.

## Conclusion

In present research, we characterize FITM1 as both a methylation-driven gene and tumor suppressor gene. Based on the investigation of 101 non-viral HCC patients in TCGA, we demonstrate that the hypermethylated FITM1 down-regulates the corresponding FITM1 expression, thereby promoting the progression of non-viral HCC *via* cancer-related pathways. On the other hand, the results also suggest that the signature composed of three methylation-driven genes can function as the prognostic indicator for non-viral HCC patients. In all, not only are potential targets and epigenetic biomarkers discovered and illustrated in our work, a FITM1-related risk signature for non-viral HCC patients is built.

## Data Availability Statement

The datasets generated for this study can be found in the TCGA database (http://www.tcga.org).

## Ethics Statement

The mRNA expression and methylation data of non-viral HCC provided by TCGA is public and the approval of a local ethics committee is unneeded.

## Author Contributions

YC and TC designed the research. JC and XicW performed the specific data-processing procedures and wrote the manuscript. XinW and WL made the pictures and graphs. CS revised the final manuscript. All the authors have read and approved the manuscript. The authors declare no conflicts of interest.

## Funding

This work was supported by the Science and Technology Program of Guangzhou, China (STPG; 2016201604030054) and the National Natural Science Foundation of China (Nos. 81800560).

## Conflict of Interest

The authors declare that the research was conducted in the absence of any commercial or financial relationships that could be construed as a potential conflict of interest.

## Supplementary Material

Table S1 Clinicopathological characteristics of 101 non-viral HCC patients obtained from TCGA.

Table S2 Detailed information of 137 MDGs figured out by MethylMix algorithm.

Table S3 Gene Ontology (GO) analysis results of 137 DEGs.

Table S4 KEGG analysis results of 137 DEGs.

Table S5 Disease Ontology (DO) analysis results of 137 DEGs.

Table S6 GSEA results based on the mRNA level of FITM1.

Table S7 GSEA results based on the methylation level of FITM1.

Table S8 GSEA analysis of Clusters classified by ConsensusClusterPlus analysis.

Figure S1 The additional results of ConsensusClusterPlus analysis and PCA analysis.

Figure S2 The analysis of FITM1 in all HCC patients in TCGA based on the GEPIA database (http://gepia.cancer-pku.cn).
