# Supplementary material for: A FITM1-Related Methylation Signature Predicts the Prognosis of Patients With Non-Viral Hepatocellular Carcinoma
Source: Front Genet. 2020 Feb 27;11:99. doi: 10.3389/fgene.2020.00099 (PMC7056874; doi:10.3389/fgene.2020.00099)
Supplement: Table S1 — Clinicopathological characteristics of 101 non-viral HCC patients obtained from TCGA. [file Table_1.docx]

**Table S1 Clinicopathological characteristics of 101 non-viral HCC patients.**

| Clinicopathological characteristics | Number of cases(%) |
| --- | --- |
| **Age (years)** |  |
| ≤ 50 | 14(14%) |
| > 50 | 87(86%) |
| **Gender** |  |
| Male | 53(52%) |
| Female | 48(48%) |
| **Race** |  |
| Asian | 16(16%) |
| Black | 4(4%) |
| White | 77(77%) |
| Not Available | 2(2%) |
| Unknown | 2(2%) |
| **Child-Pugh** |  |
| A | 47(47%) |
| B | 4(4%) |
| Not Available | 27(27%) |
| Unknown | 23(23%) |
| **Cirrhosis** |  |
| Yes | 19(19%) |
| No | 28(28%) |
| Not Available | 35(35%) |
| Unknown | 19(19%) |
| **AFP (μg/L)** |  |
| ≤ 400 | 49(49%) |
| > 400 | 13(13%) |
| Unknown | 39(39%) |
| **TNM stage** |  |
| I | 41(41%) |
| II | 21(21%) |
| III | 22(22%) |
| IV | 2(2%) |
| Unknown | 15(15%) |
| **Vascular invasion** |  |
| Yes(Macro and Micro) | 35(35%) |
| No | 57(57%) |
| Not Available | 5(5%) |
| Unknown | 4(4%) |
| **Histological grade** |  |
| G1 | 15(15%) |
| G2 | 55(55%) |
| G3 | 28(28%) |
| G4 | 1(1%) |
| Unknown | 2(2%) |
