# Supplementary figures and images for: A FITM1-Related Methylation Signature Predicts the Prognosis of Patients With Non-Viral Hepatocellular Carcinoma
Source: Front Genet. 2020 Feb 27;11:99. doi: 10.3389/fgene.2020.00099 (PMC7056874; doi:10.3389/fgene.2020.00099)

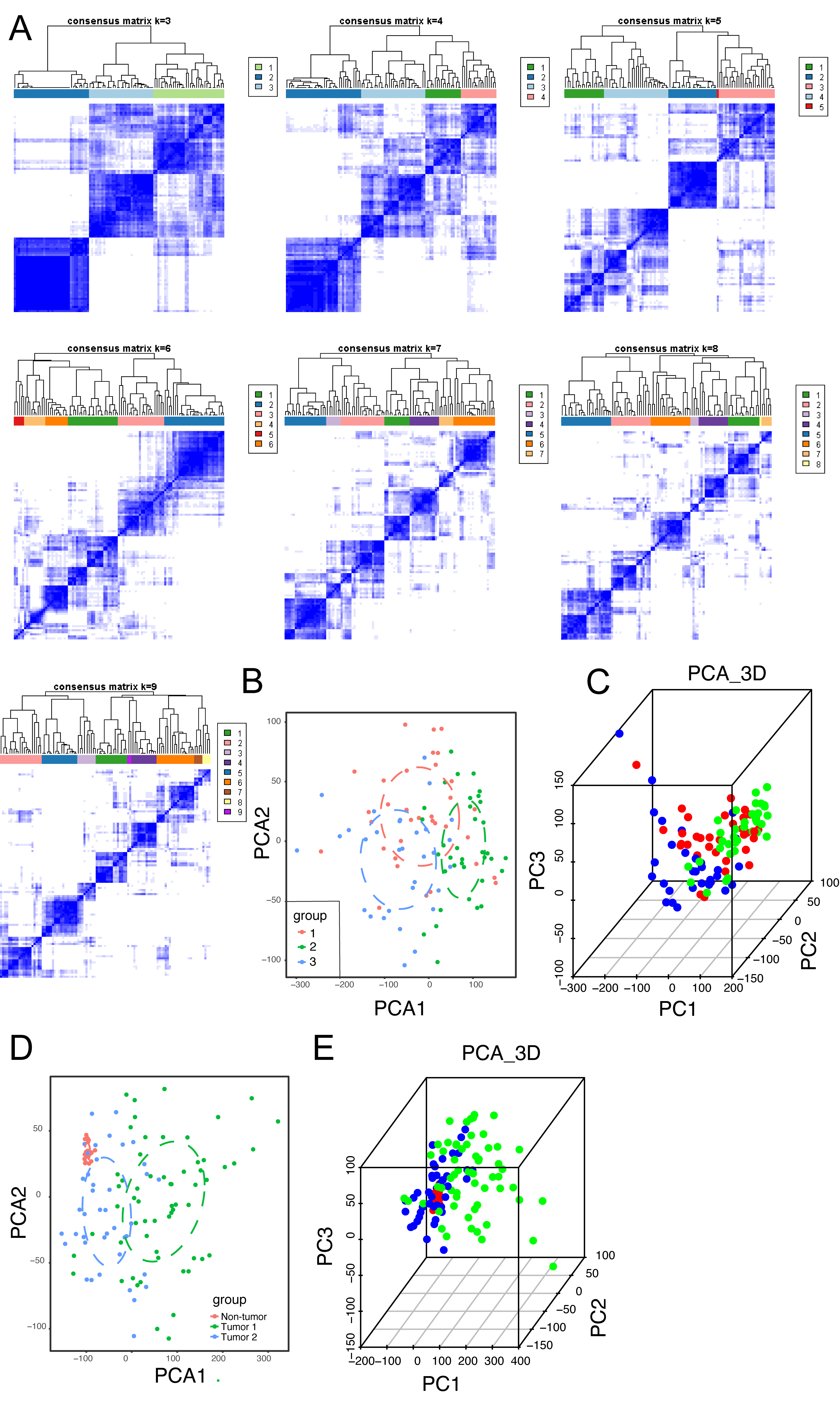

Supplement: Figure S1 — The additional results of ConsensusClusterPlus analysis and PCA analysis. [file Image_1.tif]

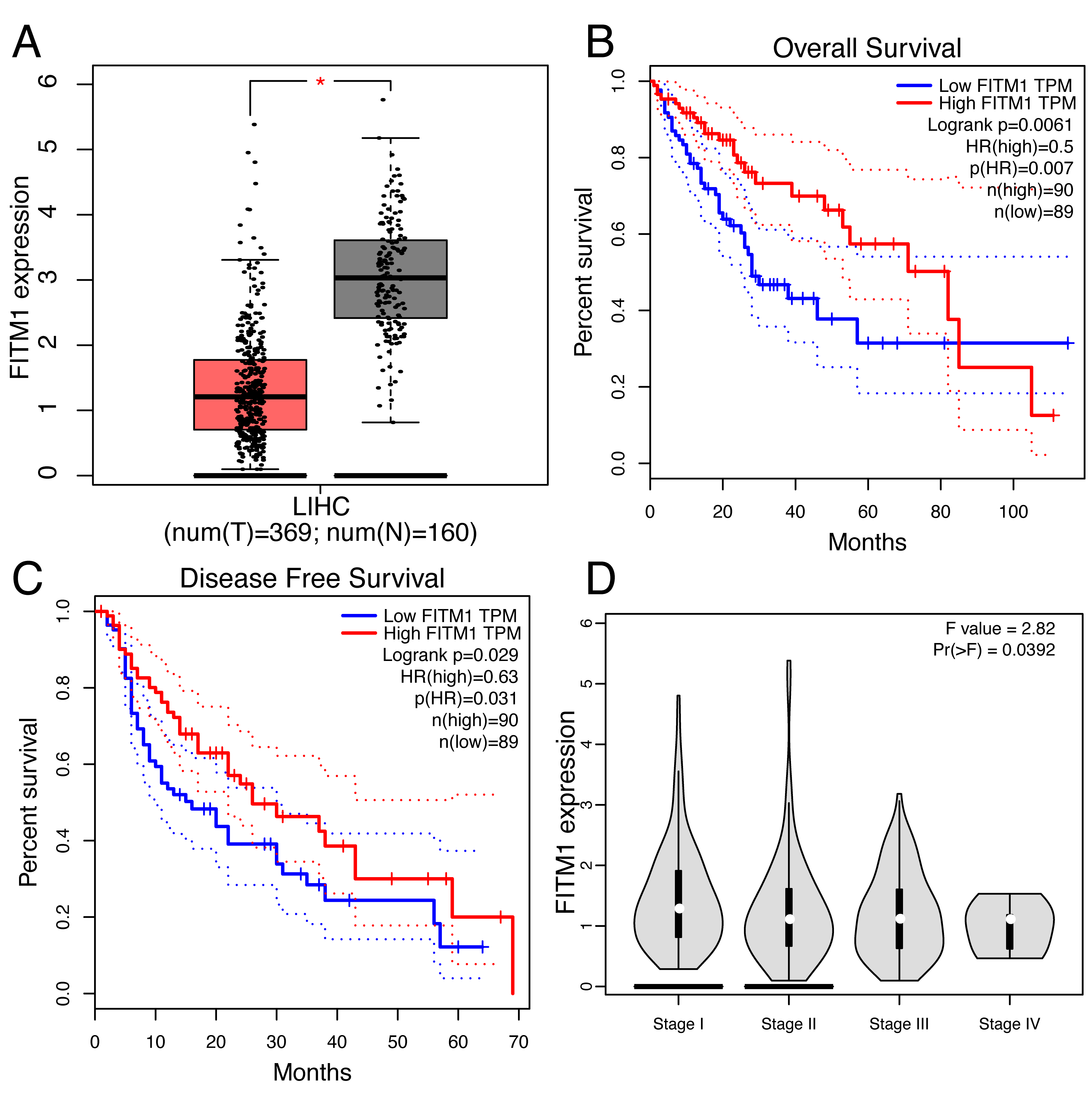

Supplement: Figure S2 — The analysis of FITM1 in all HCC patients in TCGA based on the GEPIA database (http://gepia.cancer-pku.cn). [file Image_2.tif]
